# Partial‐body cryotherapy (−135°C) and cold‐water immersion (10°C) after muscle damage in females

**DOI:** 10.1111/sms.13593

**Published:** 2019-11-27

**Authors:** Erich Hohenauer, Joseph T. Costello, Tom Deliens, Peter Clarys, Rahel Stoop, Ron Clijsen

**Affiliations:** ^1^ Department of Business Economics, Health and Social Care University of Applied Sciences and Arts of Southern Switzerland Landquart Switzerland; ^2^ International University of Applied Sciences THIM Landquart Switzerland; ^3^ Department of Movement and Sport Sciences Vrije Universiteit Brussel Brussels Belgium; ^4^ School of Sport, Health & Exercise Science University of Portsmouth Portsmouth UK

**Keywords:** cardiovascular, cold, sex, sexual dimorphism, women

## Abstract

This randomized controlled trial examined the effects of cold‐water immersion (CWI), partial‐body cryotherapy (PBC), or a passive control (CON) on physiological and recovery variables following exercise‐induced muscle damage (EIMD, 5 × 20 drop jumps) in females. Twenty‐eight females were allocated to PBC (30 seconds at −60°C, 2 minutes at −135°C), CWI (10 minutes at 10°C), or CON (10 minutes resting). Muscle oxygen saturation (SmO_2_), cutaneous vascular conductance (CVC), mean arterial pressure (MAP), and local skin temperature were assessed at baseline and through 60 minutes (10‐minute intervals), while delayed onset of muscle soreness (DOMS), muscle swelling, maximum voluntary isometric contraction (MVIC), and vertical jump performance (VJP) were assessed up to 72 hours (24‐hour intervals) following treatments. SmO_2_ was lower in PBC (Δ‐2.77 ± 13.08%) and CWI (Δ‐5.91 ± 11.80%) compared with CON (Δ18.96 ± 1.46%) throughout the 60‐minute follow‐up period (*P* < .001). CVC was lower from PBC (92.7 ± 25.0%, 90.5 ± 23.4%) and CWI (90.3 ± 23.5%, 88.1 ± 22.9%) compared with CON (119.0 ± 5.1 and 116.1 ± 6.6%, respectively) between 20 and 30 minutes (*P* < .05). Mean skin temperature was lower from CWI vs PBC (between 10 and 40 minutes, *P* < .05). Mean skin temperature was higher in CON compared with CWI up to 60 minutes and compared with PBC up to 30 minutes (*P* < .05). DOMS was lower following both PBC and CWI compared with CON through 72‐hour (*P* < .05), with no difference between groups. No main group differences for swelling, MVIC, and VJP were observed. In conclusion, CWI elicited generally greater physiological effects compared with PBC while both interventions were more effective than CON in reducing DOMS in females, but had no effect on functional measures or swelling.

## 1 INTRODUCTION

The use of cryotherapy to reduce the effects of exercise‐induced muscle damage (EIMD) is popular in the field of sport science, although evidence is limited for its effectiveness. The proposed mechanism by which cold exposure enhances recovery is attributed to its vasoconstrictive effect,^1^ and subsequent reduction of inflammation and metabolism. Subjective and objective recovery variables, used to quantify recovery of EIMD, are delayed onset of muscle soreness (DOMS), reduced maximum voluntary isometric contraction (MVIC), vertical jump performance (VJP), and muscle swelling.^2^, ^3^ Non‐invasive measures such as muscle oxygen saturation (SmO_2_), cutaneous vascular conductance (CVC), mean arterial pressure (MAP), and skin temperature have been used to explain the possible blood flow and temperature‐induced physiological effects of cooling on recovery.^4^, ^5^, ^6^

Cold‐water immersion (CWI) is a commonly employed post‐exercise recovery modality enhancing mitochondrial biogenesis after endurance training^7^, ^8^ but may attenuate muscle adaptions following strength training.^7^ Partial‐body cryotherapy (PBC) and whole‐body cryotherapy (WBC) are increasing in popularity for their use in recovery of performance; however, there remains equivocal evidence for a positive effect on WBC on functional recovery.^2^, ^3^, ^9^ With only minimal evidence supporting improvements of performance following WBC,^10^ individuals are exposed to vaporized liquid nitrogen in a head‐free cabin system during PBC, while during WBC, individuals are exposed to cold air in a closed chamber system. On the other hand, individuals are submerged in water to varying levels and at various temperatures and durations during CWI treatment. This variability in application of each of the cryotherapy modalities, CWI vs WBC vs PBC differs, which would have different physiological effects and could explain the differing results of previous studies.

Despite the ongoing debate regarding which cold treatment is the most efficient to accelerate athletic recovery, only limited evidence is available to directly compare these popular interventions. Abaidia et al (2016) were the first to compare the effects between PBC (3 minutes at −110°C) and CWI (10 minutes at 10°C) after a hamstring damaging exercise in a male population.^11^ The authors observed a moderate effect in favor of CWI for single‐leg and double‐leg jump performance after 72‐hour post‐exercise. Furthermore, CWI elicited a greater reduction in soreness 48‐hour post‐exercise compared with PBC. In contrast, Wilson et al (2017) compared the effects between WBC (3 minutes at −85°C, followed by a 15‐minute resting period under ambient environmental conditions, followed by 4 minutes at −85°C) and CWI (10 minutes at 8°C) following a marathon in healthy males. These authors only observed a trivial effect in MVIC recovery between 24 and 48 hours after the CWI treatment, and WBC was reported to have a negative effect on MVIC at 48‐hour post‐exercise.^12^ In a follow‐up study, Wilson et al (2018) observed that WBC, using a similar cooling protocol, was more effective compared with CWI (10 minutes at 10°C) to attenuate perceptual and functional recovery following resistance training in males.^13^ However, the mechanism of muscle damage between the aforementioned studies all varied, with two studies employing primarily mechanical damage (Abaidia et al 2016; Wilson et al 2018) while the other study consisted of primarily metabolic damage (Wilson et al 2017). Only two studies have investigated hemodynamic responses between CWI and PBC^14^ or CWI and WBC.^5^ Ultimately, these findings highlight the conflicting and inconsistent evidence in this field.

To our knowledge, no study has examined the physiological responses or the recovery between PBC, CWI, and a control treatment in females. This is consistent with the significantly under‐represented female participants in the wider sport and exercise medicine literature.^15^ Furthermore, a recent Cochrane review on WBC concluded that the existing literature on cryotherapy may not be applicable to females and future research on female participants is warranted in this area.^3^ Furthermore, altered hormonal status during the menstrual cycle (eg, estrogen) and the anthropometric differences (eg, fat distribution and total amount of body fat) could lead to differences after a muscle‐damaging protocol in females.

Accordingly, the aim of this study was to examine (a) the physiological effects and (b) the subjective and objective recovery characteristics of PBC (30 seconds at −60°C, 2 minutes at −135°C), CWI (10 minutes at 10°C) or a control group (CON) following EIMD in healthy, recreationally trained females. It was hypothesized that, compared to PBC or the control treatment, CWI would elicit a greater physiological effect. We further hypothesized that recovery would be quicker following CWI compared with PBC and CON.

## 2 MATERIALS AND METHODS

### 2.1 Participants

Using data from studies employing similar methodological designs,^2^, ^14^ the sample size was determined using G*Power (version 3.1.9.2; Franz Faul University Kiel, Germany). The following design specifications were taken into account: α = 0.05; power = 0.8; effect size = 0.4; statistical test = repeated measures ANOVA with within‐between interaction. The sample size estimated according to these specifications were nine participants per group. A total of thirty healthy females (age: 22.5 ± 2.7 years) were recruited for this study (Table 1); two participants in the CON group did not complete the study due to illness (unrelated to the study, n = 2).

**Table 1:** Descriptive characteristics of the female participants

Parameters	PBC (n = 10)	CWI (n = 10)	CON (n = 8)	*P*‐value
Age (years)	22.4 ± 3.0	21.9 ± 2.0	23.3 ± 2.6	.43
Height (cm)	166.7 ± 5.6	165.0 ± 8.5	168.1 ± 2.5	.20
Mass (kg)	62.8 ± 7.3	60.3 ± 3.7	63.8 ± 8.6	.37
Body fat %	32.7 ± 3.2	31.1 ± 2.9	32.0 ± 5.6	.70
BMI (kg^.^m^2^)	22.6 ± 2.3	22.2 ± 2.0	22.6 ± 3.2	.89
∑ 9 SF (mm)	145.6 ± 35.4	135.8 ± 33.6	145.9 ± 36.1	.70
BSA (m^2^)	1.7 ± 0.1	1.6 ± 0.1	1.7 ± 0.1	.32
BSA: mass (m^2^ kg^−1^)	0.027 ± 0.001	0.027 ± 0.001	0.027 ± 0.002	.80
Endomorphy	4.6 ± 1.1	4.4 ± 1.4	4.5 ± 1.5	.87
Mesomorphy	4.4 ± 1.0	3.9 ± 1.2	4.5 ± 1.8	.59
Ectomorphy	2.2 ± 1.1	2.3 ± 1.4	2.4 ± 1.4	.89

Note

Values are means ± SD.

Abbreviation: BMI, body mass index, BSA, body surface area; ∑ 9 SF, sum of 9 skinfolds.

All participant were recreationally trained (physically active for at least 2 h week^−1^), but did not have a history of resistance training. Participants were excluded from the study if they were smokers, had an allergy to cold, a history of any cardiovascular or respiratory disease, any existing symptoms of pain, or were taking medication (excluding oral contraception). The participants were instructed to refrain from alcohol, supplements, and exercise during the experimental period. All included participants were fully informed about the aims and risks of this study, as well as the discomforts related to this study, before signing an informed consent form. This study was approved by the Ethical Committee of Zurich and is registered in the http://clinicaltrials.gov registry (NCT02847663).

### 2.2 Experimental design

This study employed a randomized controlled, parallel group design. The methodological design has previously been advocated in EIMD research.^3^ The study was completed over five experimental days, with testing taking place at the same time of day to minimize any potential effects of circadian rhythm. On day one, participants were familiarized to the VJP and MVIC procedure. On experimental day two (7‐days after day 1), participants were randomly allocated into either the PBC, CWI, or CON group. Anthropometric characteristics were assessed from an ISAK qualified practitioner and baseline data were recorded. The datasets of the n = 2 drop‐outs were excluded from the entire analyses.

**Figure 1:**
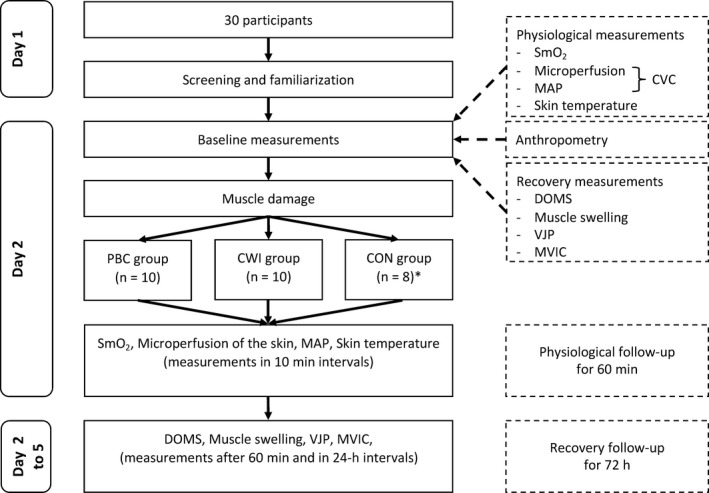
Schematic representation of the experimental protocol in function of time. CON, control; CVC, cutaneous vascular conductance; CWI, cold‐water immersion; DOMS, delayed onset of muscle soreness; MAP, mean arterial pressure; MVIC, maximum voluntary isometric contraction; PBC, partial‐body cryotherapy; SmO_2_, muscle oxygen saturation; VJP, vertical jump performance. *n = 2 drop‐outs due to illness

The physiological variables measured were SmO_2_, CVC, MAP, and skin temperature. These variables were always assessed in supine position before (baseline), after (0 minute) and at 10‐min intervals (up to 60 minute) after the treatments. The recovery variables measured were DOMS, muscle swelling, VJP, and MVIC. These variables were assessed before (baseline), after 60‐min (1 hour) and in 24‐hour intervals (up to 72 hours) after the treatments on day 2‐5, always in the aforementioned order. All variables were assessed by the same investigator who was not blinded to the recovery interventions. The environmental conditions were kept constant over the five experimental days (room temperature: 21 ± 2°C, relative room humidity: 45 ± 5%). A schematic representation of the test protocol is presented in Figure 1.

### 2.3 Induction of muscle damage

A validated protocol described in detail elsewhere was used to induce muscle damage in the knee extensor muscles.^16^ Briefly, participants performed 100 drop jumps from a 0.6 m box (5 sets of 20 drop jumps with a 2‐minute break between the sets). The participants were verbally encouraged and instructed to jump up maximally upon landing. After all baseline measurements were completed, a brief explanation and demonstration of the drop‐jump protocol were provided to the participants. Thereafter, the participants performed the self‐paced (approximately 20 minutes) muscle‐damaging exercise, which was followed immediately by the recovery interventions.

### 2.4 Recovery interventions

The PBC group entered the cryocabin (Cryomed sro., Cryosauna Space Cabin, Nové Zámky, Slowakia) and was exposed to vaporized liquid nitrogen for 30 seconds at −60°C and then for 2 minutes at −135°C as previously described.^17^ All participants wore bikinis and cold‐resistant shoes. The participants placed their hands on the edge of the cabin and turned around the y‐axis, as described in the user's manual.^14^

The CWI group was submerged in a plastic tub (height: 85 cm, diameter: 75 cm) to the level of the sternum in stirred cold water (10°C) for 10 minutes, which is the most common CWI protocol used in the literature.^18^ The temperature of water was monitored with a thermometer throughout (Voltacract MT52, Wollerau, Switzerland). After the immersion protocol, the participants toweled dry and changed into dry bikinis.

The CON group received no treatment and rested in bikinis in a supine position, for the duration of 10 minutes (room temperature: 21 ± 2°C, relative room humidity: 45 ± 5%).

### 2.5 Physiological measurements

#### 2.5.1 Muscle oxygen saturation

SmO_2_ of the knee extensor muscle (vastus lateralis) was measured using near‐infrared spectroscopy (Moxy, Swinco). This technique has been previously demonstrated as a valid and reliable method of measuring muscle oxygenation.^19^ The monitor was placed on the right vastus lateralis, midway between the proximal patella, and the inguinal crease.^4^ Due to technical and safety reasons, the monitor was removed during the EIMD protocol and during the PBC and CWI treatments. The location of the monitor was marked using a permanent marker and reaffixed to the same anatomical site after the treatments.

#### 2.5.2 Cutaneous vascular conductance

The microperfusion of the left anterior thigh was measured with a Laser‐Speckle Contrast Imaging (LSCI) device (moorFLPI2, Moor instruments. Millwey).^20^ The participants were instructed to shave their legs 24 hours prior to the experiment and a priori, a 21 cm^2^ area of interest was marked to ensure reliable measurements. Due to its high spatial resolutions, the LSCI device is capable of recording rapid changes in superficial blood flow across a larger skin surface area.^21^ CVC was calculated as the ratio between the LSCI flux and MAP, as previously described.^5^, ^14^

#### 2.5.3 Mean arterial pressure

Blood pressure was measured using an automated sphygmomanometer monitor (Microlife BP 3 BTO‐AP) from the left brachial artery. MAP was calculated using the following formula^22^:

MAP=diastolic blood pressure+(systolic blood pressure-diastolic blood pressure)/3

#### 2.5.4 Skin temperature

Local skin temperature at the neck (*T*_neck_), right infraspinous fossa (*T*_scapula_), right dorsal hand (*T*_hand_), right mid‐shin (*T*_shin_), and the right anterior thigh (*T*_thigh_), 2 cm above the SmO_2_ monitor, were recorded using iButtons (iButton, Maxim Integrated).^23^ In accordance to ISO 9886, the mean skin temperature was calculated from four sites using the following formula^24^:

$$\text{Mean skin temperature}=\left(T_{\text{neck}}*0.28\right)+\left(T_{\text{scapula}}*0.28\right)+\left(T_{\text{hand}}*0.16\right)+\left(T_{\text{shin}}*0.28\right)$$

### 2.6 Recovery measurements

#### 2.6.1 Delayed onset of muscle soreness

Subjective ratings of knee extensor muscle soreness were assessed using a 0‐10 cm visual analogue scale.^25^ The far‐left endpoint 0 indicated “no soreness” while the far‐right endpoint 10 indicated “severe muscle soreness.” The participants were instructed to rate their level of soreness during a squat, which was maintained isometrically for 3 seconds (90° knee angle), as previously described.^25^

#### 2.6.2 Muscle swelling

Swelling of the right anterior thigh was assessed by the same investigator, via ultrasound (MyLabClassC, Esaote) in a supine position. The skin was marked with a permanent marker at 60% of the distance between the greater trochanter and lateral epicondyle and 3 cm lateral to the midline of the anterior thigh as previously described.^2^ The ultrasound probe was placed, without compression, on water‐soluble gel. Muscle swelling was defined as the distance from the muscle‐bone interface to the subcutaneous adipose tissue‐muscle interface.

#### 2.6.3 Vertical jump performance

Vertical jump performance was measured on a jump plate (Just Jump, Probotics Inc). The participants had to perform standardized countermovement jumps, with their hands placed on their hips, as described previously.^26^ The participants were instructed to jump as high as possible, were not verbally encouraged during the jump performance, and were blinded to the VJP values. VJP was measured three times in a row with a 1 minute break between each set. The maximum value of these three attempts was used to assess VJP on each experimental day.

#### 2.6.4 Maximum voluntary isometric contraction

Maximum voluntary isometric contraction of the right knee extensor muscle was measured on an ergometer chair (Cor‐1, V.1.0., OT Bioelettronica) at a knee angle of 120° and a hip angle of 100° as previously described.^14^ The participants’ right shin was strapped to the chair to ensure an isometric contraction. Then, participants were instructed to maximally extend their knee for the duration of 4 seconds and were not verbally encouraged. MVIC was measured three times in a row with a 2‐minute break between each set. The maximum value of these three attempts was used to assess MVIC on each experimental day. The participants were blinded to the MVIC values.

### 2.7 Data analysis

Descriptive results are reported as means ± standard deviations (SD). Assumption of normality was verified using the Shapiro‐Wilk test. The physiological and recovery variables were analyzed using repeated measures ANOVAs mixed design with treatment (PBC, CWI, and CON) as between factor, and time (for physiological measurements: baseline, 0, 10, 20, 30, 40, 50, 60 minutes; for recovery measurements: baseline, 1, 24, 48, 72 hours) as within factor (see Figure 1). Post‐hoc analyses using Bonferroni correction were performed where appropriate. One‐way ANOVAs with Tukey corrected post‐hoc analyses were used to evaluate the differences between PBC, CWI, and CON per time point (baseline, 0, 10, 20, 30, 40, 50, 60 minutes or baseline, 1, 24, 48, 72 hours). Effect size was expressed as partial eta squared ($\eta_{\text{partial}}^{2}$) values, with 0.01, 0.06, and 0.14 being considered as small, medium, and large, respectively.^27^ All statistical analyses were performed using the statistical package for the social sciences (SPSS Inc), version 24.0 with the level of significance set *P* < .05.

## 3 RESULTS

The absolute baseline values of the physiological and recovery variables are displayed in Table 2.

**Table 2:** Absolute baseline values for all variables

Parameters	PBC (n = 10)	CWI (n = 10)	CON (n = 8)
SmO_2_ (%)	87.2 ± 3.9	87.0 ± 6.7	80.1 ± 1.8
CVC (flux [AU].MAP [mm Hg]^−1^	0.8 ± 0.2	0.7 ± 0.4	0.4 ± 0.04
MAP (mm Hg)	89.8 ± 10.2	91.4 ± 9.6	82.3 ± 3.3
DOMS (cm)	0.0 ± 0.0	0.0 ± 0.0	0.0 ± 0.0
Muscle thickness QFM (cm)	3.6 ± 1.2	3.2 ± 0.3	3.3 ± 0.3
MVIC (N)	467.9 ± 86.7	428.2 ± 70.1	334.7 ± 83.4
VJP (cm)	37.5 ± 3.2	38.4 ± 3.8	36.3 ± 3.6

Note

Values are means ± SD.

Abbreviations: AU, arbitrary units; CVC, cutaneous vascular conductance; DOMS, delayed onset of muscle soreness; MAP, mean arterial pressure; mmHg, millimeters of mercury; MVIC, maximum voluntary isometric contraction; N, newton; QFM, quadriceps femoris muscle; SmO_2_, muscle oxygen saturation; VJP, vertical jump performance.

### 3.1 Physiological measurements

#### 3.1.1 Muscle oxygen saturation

**Figure 2:**
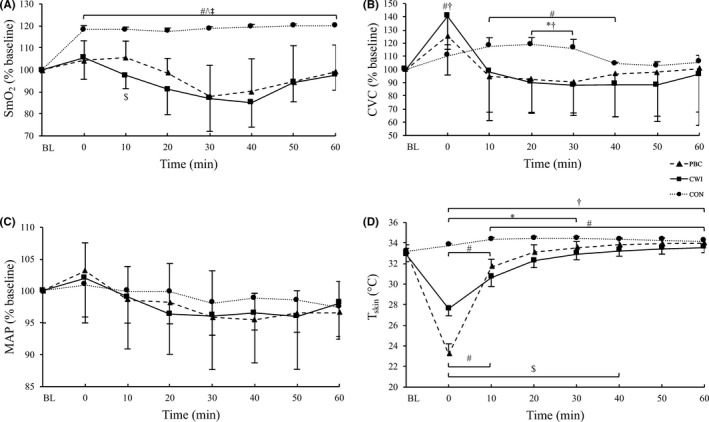
Results of (A) muscle oxygen saturation of the right vastus lateralis of the quadriceps femoris muscles (SmO_2_), (B) cutaneous vascular conductance (CVC), (C) mean arterial pressure (MAP), and (D) mean skin temperature (*T*_skin_) in function of time. Values for A, B, and C are normalized to baseline (% mean ± SD) with respect to their initial values. BL baseline, ^#^ *P* < .05 compared to baseline, ^$^ *P* < .05 PBC vs CWI, ^*^ *P* < .05 PBC vs CON, ^†^ *P* < .05 CWI vs CON, ^^^ *P* < .001 PBC vs CON, ^‡^ *P* < .001 CWI vs CON

For SmO_2_, a significant treatment effect (*F*_2,25_ = 30.46, *P* < .001, $\eta_{\text{partial}}^{2}$ = 0.70), time effect (*F*_7,19_ = 16.2, *P* < .001, $\eta_{\text{partial}}^{2}$ = 0.85), and treatment*time interaction (*F*_14,40_ = 6.83, *P* < .001, $\eta_{\text{partial}}^{2}$ = 0.70) were observed (Figure 2A). Figure 2 depicts the reductions in SmO_2_ after both PBC and CWI and a concurrent increase in the CON group. SmO_2_ was significantly lower in the CWI group compared with the PBC group 10 minutes after the treatments (absolute values, normalized to baseline values; PBC 10 minutes: 91.8 ± 3.8%, 105.6 ± 7.4% vs CWI 10 minutes: 85.0 ± 10.1%, 97.4 ± 5.9%, *P* = .01). Both PBC and CWI values were significantly lower compared with values in the CON group throughout the 60‐min follow‐up period (all *P* < .001).

#### 3.1.2 Cutaneous vascular conductance

Despite no significant treatment effect (*F*_2,25_ = 1.08, *P* = .35, $\eta_{\text{partial}}^{2}$ = 0.08), a significant time (*F*_7,19_ = 7.39, *P* < .001, $\eta_{\text{partial}}^{2}$ = 0.73) and treatment*time interaction (*F*_14,40_ = 2.95, *P* = .004, $\eta_{\text{partial}}^{2}$ = 0.50) were observed in CVC (Figure 2B). CVC decreased over time in the cold groups but increased in the CON group. No differences were observed between PBC and CWI at any time point. However, CVC values were significantly lower in the PBC vs the CON group after 20 minutes (PBC 20 minutes: 0.80 ± 0.33 flux.MAP^−1^, 92.7 ± 25.0% vs CON 20 minutes: 0.59 ± 0.04 flux.MAP^−1^, 119.0 ± 5.1%, *P* = .03) and 30 minutes (PBC 30 minutes: 0.77 ± 0.30 flux.MAP^−1^, 90.5 ± 23.4% vs CON 30 minutes: 0.55 ± 0.05 flux.MAP^−1^, 116.1 ± 6.6%, *P* = .02). CVC was also lower in the CWI compared with the CON group between 20 minutes (CWI 20 minutes: 0.71 ± 0.44 flux.MAP^−1^, 90.3 ± 23.5% vs CON 20 minutes: 0.59 ± 0.04 flux.MAP^−1^, 119.0 ± 5.1%, *P* = .004) and 30 minutes (CWI 30 minutes: 0.75 ± 0.45 flux.MAP^−1^, 88.1 ± 22.9% vs CON 30 minutes: 0.55 ± 0.05 flux.MAP^−1^, 116.1 ± 6.6%, *P* = .003) and significantly higher in the CWI compared with the CON group immediately after the treatment (CWI 0 minute: 1.22 ± 0.78 flux.MAP^−1^, 140.3 ± 25.0% vs CON 0 minute: 0.52 ± 0.05 flux.MAP^−1^, 110.6 ± 8.1%, *P* = .02).

#### 3.1.3 Mean arterial pressure

No significant treatment effect (*F*_2,25_ = 0.23, *P* = .79, $\eta_{\text{partial}}^{2}$ = 0.01) or time*treatment interaction (*F*_14,40_ = 1.45, *P* = .17, $\eta_{\text{partial}}^{2}$ = 0.33) were observed for MAP (Figure 2C). However, a significant reduction over time (*F*_7,19_ = 11.97, *P* < .001, $\eta_{\text{partial}}^{2}$ = 0.81) was evident.

#### 3.1.4 Skin temperature

A significant treatment effect (*F*_2,25_ = 57.23, *P* < .001, $\eta_{\text{partial}}^{2}$ = 0.82), time effect (*F*_7,19_ = 293.37, *P* < .001, $\eta_{\text{partial}}^{2}$ = 0.99), and time*treatment interaction were observed (*F*_14,40_ = 28.3, *P* < .001, $\eta_{\text{partial}}^{2}$ = 0.90) for the mean skin temperature (Figure 2D). In the PBC and CWI group, mean skin temperature decreased over time, while it increased in the CON group. Mean skin temperature was significantly lower in the CWI group compared with the PBC group between 10 and 40 minutes (all *P* < .05). Mean skin temperature was lower in the PBC group compared with the CON group up to 30 minutes (*P* < .05 for all differences) and compared with CWI only after the treatment (0 minute: PBC: 23.1 ± 1.0°C vs CWI: 27.6 ± 0.6°C, *P* < .001). CWI resulted in significantly lower values compared with the values in the CON group up to 60 minute after the treatment (*P* < .05 for all differences). The results for the local skin temperatures are presented in Table 3.

**Table 3:** Local skin temperature data

	Baseline	0 min	10 min	20 min	30 min	40 min	50 min	60 min
T_thigh_ (°C)		e, f	d, e, f	b, d, f	a, f	a, f	c	c
PBC	31.0 ± 1.07	15.3 ± 2.2	28.6 ± 0.8	30.2 ± 1.0	30.8 ± 1.0	31.1 ± 1.0	31.2 ± 1.0	31.4 ± 1.0
CWI	31.40 ± 0.7	14.8 ± 1.4	24.9 ± 0.9	27.8 ± 1.1	29.1 ± 1.1	29.8 ± 1.0	30.3 ± 1.0	30.6 ± 1.0
CON	31.3 ± 0.3	30.9 ± 0.1	31.5 ± 0.2	31.7 ± 0.3	31.8 ± 0.3	31.9 ± 0.2	32.0 ± 0.2	32.1 ± 0.1
T_shin_ (°C)	^ ^	e, f	d, e, f	d, e, f	b, d, f	d, f	d, f	d, f
PBC	32.0 ± 0.9	14.4 ± 1.4	29.9 ± 0.6	31.3 ± 0.5	31.8 ± 0.4	32.0 ± 0.4	32.1 ± 0.5	32.1 ± 0.5
CWI	32.2 ± 0.7	15.2 ± 1.3	26.0 ± 0.9	28.5 ± 0.6	29.3 ± 0.5	29.8 ± 0.4	30.1 ± 0.4	30.2 ± 0.4
CON	32.1 ± 0.1	32.8 ± 0.3	33.0 ± 0.2	32.7 ± 0.1	32.4 ± 0.2	32.1 ± 0.1	31.8 ± 0.1	31.6 ± 0.2
T_neck_ (°C)		c, d, e	c, e	c				
PBC	33.8 ± 0.7	28.4 ± 1.4	32.9 ± 1.1	34.3 ± 1.3	34.6 ± 1.4	34.9 ± 1.6	35.2 ± 1.6	35.4 ± 1.5
CWI	33.6 ± 0.7	33.0 ± 0.9	32.7 ± 1.0	33.9 ± 1.3	34.5 ± 1.2	35.0 ± 1.0	35.3 ± 1.0	35.4 ± 1.0
CON	33.2 ± 0.2	34.5 ± 0.2	35.1 ± 0.4	35.5 ± 0.4	35.7 ± 0.4	35.8 ± 0.5	35.9 ± 0.4	36.0 ± 0.4
T_scapula_ (°C)		d, e, f	e, f	e, f	a, b		a	a
PBC	33.6 ± 1.0	24.7 ± 1.7	32.6 ± 1.1	34.5 ± 1.1	35.1 ± 0.8	35.7 ± 0.6	36.0 ± 0.5	36.1 ± 0.5
CWI	33.4 ± 0.9	32.5 ± 1.2	32.9 ± 1.1	35.0 ± 0.8	35.8 ± 0.6	36.1 ± 0.6	36.4 ± 0.4	36.6 ± 0.3
CON	34.2 ± 0.2	35.2 ± 0.3	35.9 ± 0.2	36.1 ± 0.2	36.2 ± 0.2	36.2 ± 0.2	36.3 ± 0.1	36.4 ± 0.2
T_hand_ (°C)		d, e		c	c		c	
PBC	32.3 ± 1.2	26.4 ± 1.0	31.4 ± 1.3	32.2 ± 1.2	32.0 ± 1.0	31.7 ± 1.0	31.4 ± 1.0	31.0 ± 1.0
CWI	32.0 ± 1.6	31.2 ± 1.9	30.9 ± 1.9	31.1 ± 1.5	31.0 ± 1.4	30.9 ± 1.4	30.7 ± 1.3	30.6 ± 1.2
CON	32.4 ± 0.3	31.2 ± 0.2	32.4 ± 0.1	32.7 ± 0.3	32.3 ± 0.4	32.1 ± 0.5	32.0 ± 0.7	31.5 ± 0.7

Note

Values are means ± SD.

Abbreviations: CON, control; CWI, cold‐water immersion; PBC, partial‐body cryotherapy.

a *P* < .05 between PBC and CWI.

b *P* < .05 between PBC and CON.

c *P* < 0.05 between CWI and CON.

d *P* < 0.001 between PBC and CWI.

e *P* < 0.001 between PBC and CON.

f *P* < 0.001 between CWI and CON.

### 3.2 Recovery measurements

#### 3.2.1 Delayed onset of muscle soreness

**Figure 3:**
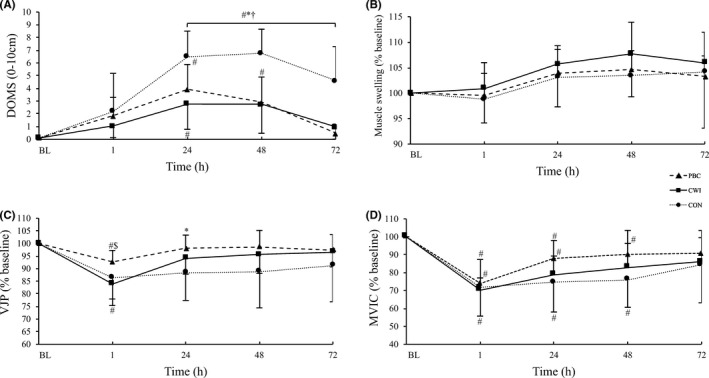
Results of (A) delayed onset of muscle soreness (DOMS), (B) muscle swelling of the right quadriceps femoris muscles, (C) vertical jump performance (VJP), and (D) maximum voluntary isometric contraction (MVIC). Values for B, C, and D are normalized to baseline (% mean ± SD) with respect to their initial values. BL baseline, ^#^ *P* < .05 compared with baseline, ^$^ *P* < .05 PBC vs CWI, ^*^ *P* < .05 PBC vs CON, ^†^ *P* < .05 CWI vs CON

A significant treatment (*F*_2,25_ = 11.30, *P* < .001, $\eta_{\text{partial}}^{2}$ = 0.47), time (*F*_4,22_ = 28.80, *P* < .001, $\eta_{\text{partial}}^{2}$ = 0.84), and time*treatment interaction (*F*_8,46_ = 3.4, *P* = .004, $\eta_{\text{partial}}^{2}$ = 0.37) were observed in DOMS (Figure 3A). In all three groups, DOMS increased over the time. DOMS was lower in the PBC and CWI group compared with the CON group throughout the 72‐hour follow‐up period (*P* < .05 for all [PBC vs CON and CWI vs CON] between group differences,). No differences between PBC and CWI were observed for DOMS.

#### 3.2.2 Muscle swelling

For muscle swelling, despite no treatment effect (*F*_2,25_ = 1.77, *P* = .19, $\eta_{\text{partial}}^{2}$ = 0.12) or time*treatment interaction (*F*_8,46_ = 0.6, *P* = .75, $\eta_{\text{partial}}^{2}$ = 0.09), a significant time effect was observed (*F*_4,22_ = 10.3, *P* < .001, $\eta_{\text{partial}}^{2}$ = 0.65; Figure 3B). Muscle swelling increased in all three groups over the time.

#### 3.2.3 Vertical jump performance

No significant treatment effect (*F*_2,25_ = 2.26, *P* = .12, $\eta_{\text{partial}}^{2}$ = 0.15), but a significant time effect (*F*_4,22_ = 27.83, *P* < .001, $\eta_{\text{partial}}^{2}$ = 0.83) and time*treatment interaction (*F*_8,46_ = 3.17, *P* = .006, $\eta_{\text{partial}}^{2}$ = 0.35) were observed for VJP (Figure 3C). VJP decreased in all three groups over the time. After 1 hour, VJP was higher following PBC compared with CWI group (absolute value, normalized to baseline value; PBC 1 hour: 34.8 ± 3.9 cm, 92.6 ± 4.5% vs CWI 1 hour: 32.2 ± 4.8 cm, 83.9 ± 5.9%, *P* = .03). VJP values were significantly higher in the PBC group after 24 hours compared with the values in the CON group (PBC 24 hours: 36.8 ± 3,6 cm, 98.1 ± 5.1% vs CON 24 hours: 32.2 ± 5.1 cm, 88.5 ± 11.1%, *P* = .01). No differences were detected between CWI and CON for VJP.

#### 3.2.4 Maximum voluntary isometric contraction

A significant time effect (*F*_4,22_ = 39.96, *P* < .001, $\eta_{\text{partial}}^{2}$ = 0.87), but no treatment effect (*F*_2,25_ = 1.27, *P* = .29, $\eta_{\text{partial}}^{2}$ = 0.09) or time*treatment interaction (*F*_8,46_ = 1.23, *P* = .30 $\eta_{\text{partial}}^{2}$ = 0.178) was observed in MVIC (Figure 3D). In all three groups, MVIC decreased over time.

## 4 DISCUSSION

This is the first study that compared the physiological responses and recovery characteristics following PBC (−60°C for 30 seconds, −135°C for 2 minutes), CWI (10°C for 10 minutes), and a passive control treatment in healthy, recreationally trained females. The main findings of this study are: (1) the physiological effects of PBC are generally similar to CWI and (2) compared with CON, DOMS improved quicker after both PBC and CWI, while limited differences in muscle swelling and strength parameters were observed between the three treatments. These data contrast our previous findings in males utilizing the same exercise and recovery intervention.

As expected, a significant increase in SmO_2_ (~20%) was observed in the CON group after exercise. This is most likely related to an exercise induced increase in vasodilatation and muscle temperature.^28^ Both PBC and CWI reduced SmO_2_ following the EIMD protocol compared with baseline by ~15%, but this decrease was not significant. Recently, we reported that CWI, following the same muscle‐damaging protocol as in the current study, significantly decreased SmO_2_ in the vastus lateralis compared with baseline and compared with a PBC treatment in male participants.^14^ Similarly, following endurance cycling exercise, greater reductions in both femoral artery and cutaneous blood flow have been demonstrated following CWI compared with WBC in males.^5^ This indicates that changes in muscle tissue oxygenation are observed in male, but not in female participants. We hypothesized that the larger amount of adipose tissue in females compared with males might be one reason for these differences.^29^ This is confirmed in the data from our previous study, in which male participants had a body fat of 17.2 ± 5.6% in the CWI and 20.6 ± 7.5% in the PBC group, respectively, while the female participants in the current study had a body fat of 31.1 ± 2.9% in the CWI and 32.7 ± 3.2% in the PBC group, respectively. Increased subcutaneous adiposity, which has low thermal conductivity creating an insulating effect,^30^ is inversely correlated with reduction in intramuscular temperature.^31^ Although we did not measure intramuscular temperatures in either study, we can speculate that the reduction in SmO_2_ in the leaner male participants might be explained by greater reductions in the superficial intramuscular temperatures. The body surface area to mass ratio of the females is also slightly higher in the present study (CWI: 0.027 ± 0.001 m^2^ kg^‐1^, PBC: 0.027 ± 0.001 m^2^ kg^−1^) compared with the males in the earlier study (CWI: 0.025 ± 0.001 m^2^ kg^−1^, PBC: 0.024 ± 0.002 m^2^ kg^−1^). Consequently, the ability to lose heat is greater in females compared with males, and coupled with a higher concentration of body fat percentage, females may have a greater insulative capacity compared with males.^32^ Therefore, a longer duration of cooling might be needed to elicit an effect in females.

In an attempt to explain these SmO_2_ findings, we performed a post‐hoc analysis to examine the correlation between Δ_max_SmO_2_ with the male and female participants anthropometric data (ie, ∑ 9 skinfolds [mm], thigh skinfold [mm], body surface area [m^2^], body surface area:mass ratio [m^2^ kg^−1^], body mass index [kg m^2^], body fat [%], and body composition [endomorphy, mesomorphy, and ectomorphy]). Surprisingly, no significant (all *P* > .05) group (females *r* ^2^ range from −0.25 to 0.42; males *r* ^2^ range from −0.24 to 0.42) or combined participant correlation (*r* ^2^ range from −0.21 to 0.20) was observed. This could be due to the underlying, inter‐ and intra‐individual physiological differences in subcutaneous anatomy and intramuscular arterioles, leading to different levels of muscle oxygenation.^33^ In contrast to our study, Mawhinney et al (2017) reported significantly reduced skin microcirculation and femoral artery conductance after CWI compared with WBC in males.^5^ These differences may be attributed to the different cryotherapy modalities (WBC vs PBC), in addition to possible differences in skin characteristics between females and males.

In agreement with the existing literature,^14^, ^34^ peripheral skin temperatures in the lower limbs were significantly reduced compared with baseline after both PBC and CWI. These findings demonstrate that skin temperature decreases to a similar level in both males^14^ and females, after PBC and CWI treatments. CWI reduces skin temperature of the lower limbs to a greater extent compared with PBC, also in a female population (Table 3). The mean skin temperature was significantly lower in the CWI group compared with the PBC group between 10 and 40 minutes post‐treatment (Figure 2D) although the neck and scapula were not affected from the cold treatment in the CWI group compared with the PBC group (Table 3). Interestingly, the skin temperature of the neck and scapula returned to baseline after 10‐minute post‐treatment in the PBC group. A possible explanation might be, that the actual temperature in the cryocabin, especially in the chest region, is higher compared with the manufacturer‐reported temperature.^35^ Mean skin temperature significantly increased in the CON group during the 60‐min follow‐up, demonstrating that the high‐intensity protocol significantly increased skin temperature.

Although DOMS values recovered earlier following both cryotherapy treatments compared with control, no main differences were observed between PBC, CWI, and CON for muscle swelling and the strength parameters. Interestingly, one hour in to recovery, VJP performance was significantly higher in the PBC compared with the CWI group. After 24 hours, VJP values were closer to baseline in the PBC group in comparison to the CON group. In line with our study results, Fonda et al (2013) observed no significant differences between PBC and CON on maximum power output in a male population during a 96‐hour recovery period.^9^ While others^2^ have demonstrated attenuated muscle swelling of the anterior thigh and improved isometric peak torque following PBC (3 min at −110°C) after 24 h in males compared to CON. The EIMD protocol was the same as used in the present study. Our results support previous findings showing that CWI appears to be effective at restoring muscle function during jump performance,^36^ indicating that CWI may be more effective for recovery of stretch‐shortening cycle movements than isometric strength recovery.^37^ Interestingly, our results indicate that PBC is more effective than CWI in restoring short‐term VJP (see Figure 3C).

This study is not without limitations. Firstly, due to logistical constraints, the stage of menstrual cycle was not controlled for. Different estrogen levels of the participants might have contributed to the different results in the present study. In animal studies, it is documented that estrogen has a sufficient protective effect on skeletal muscle tissue to attenuate the muscle‐damaging processes following exercise.^38^ In humans, the protective effect of estrogen levels and the influence of oral contraception on muscle damage are less clear,^39^ but it is likely that the protective effect of estrogen might have a significant impact on the outcome.^40^ Future studies should take these variables into account when investigating the effects of cold treatments after muscle‐damaging protocols in females. Furthermore, due to financial constraints, incorporating a range of inflammatory cytokines (eg, IL‐6, TNF‐alpha) and biomarkers of muscle damage (eg, creatine kinase, myoglobin), which would have provided further insight into the inflammatory and damage effects of these treatments, was not feasible.

## 5 PERSPECTIVES

Existing literature comparing PBC and CWI in females is limited.^3^ The current findings will be of interest to sport science practitioners and medical personnel who are considering using either PBC or CWI interventions to improve recovery. Our study is the first one that directly compared the physiological responses and effects on muscle recovery between PBC and CWI in a female population. We demonstrate that the main physiological difference found between the PBC and CWI in females is that CWI reduces skin temperature significantly compared with PBC which could explain the generally greater physiological effects of CWI in this study. Although PBC and CWI were superior to CON for reducing DOMS, no main difference between PBC, CWI, and CON was observed in muscle swelling, strength, or VJP. From an athletic recovery perspective, these findings support those of previous studies on male participants and expand our understanding of the effectiveness of PBC and CWI to healthy, recreationally trained females.
